# Primary pituitary tuberculosis

**DOI:** 10.4322/acr.2020.228

**Published:** 2020-12-08

**Authors:** Tarun Kumar, Jitendra Singh Nigam, Iffat Jamal, Vikas Chandra Jha

**Affiliations:** 1 All India Institute of Medical Science, Department of Pathology, Patna, Bihar, India.; 2 Indira Gandhi Institute of Medical Sciences, Department of Hematology, Patna, Bihar, India.; 3 All India Institute of Medical Science, Department of Neurosurgery, Patna, Bihar, India.

**Keywords:** Tuberculosis, Granuloma, Adenoma, Pituitary Gland

## Abstract

Tuberculosis is an infectious disease that involves any organ. However, the primary pituitary tuberculosis is an extremely rare disease. Intracranial tuberculomas account for 0.15-5% of intracranial space-occupying lesions, of which, pituitary as the primary site is unusual, and easily misdiagnosed as pituitary adenoma. In this setting, the late diagnosis can result in permanent endocrine dysfunction. We report the case of a 50-year-old woman who presented to the neurosurgery outpatient department with complaints of progressively increasing headache and diminished vision over the last year. On the clinical examination, the patient was conscious and oriented. The routine hematological and biochemical workup showed an increased erythrocyte sedimentation rate (ESR) and increased prolactin levels. The radiological working diagnosis was consistent with pituitary macroadenoma. No other radiological and/or clinical clue that could elicit the suspicion of pulmonary or extrapulmonary lesions of tuberculosis was found. The transsphenoidal endonasal tumor excision was done. The histopathology showed numerous epithelioid cell granulomas, Langhans giant cells along with scant necrosis. Ziehl Neelsen staining demonstrated acid-fast bacilli, and the final diagnosis of pituitary tuberculoma was made. We report this rare case of pituitary lesion that may be included in the differential diagnosis of sellar lesions to avoid unnecessary surgical interventions, especially in regions where the disease is endemic.

## INTRODUCTION

Tuberculosis (TB) is caused by *Mycobacterium tuberculosis* and is one of the major culprits for death from a solitary contagious agent globally.^1^ India accounts for approximately 25% of TB’s worldwide burden and is also one of the leading regions with many extrapulmonary TB cases.^1^ ^,^ ^2^ TB can involve any human organ or body part; however, the lungs are predominantly involved.^3^ Extrapulmonary TB can occur with or without pulmonary TB and comprises 14% of the cases reported in 2017 globally.^1^ ^,^ ^3^ ^,^ ^4^ TB of the central nervous system (CNS) constitutes only 1% of the TB cases globally and is more common in areas with high TB prevalence rate.^3^ ^,^ ^4^ CNS TB can involve the meninges, brain, or adjacent bone by the hematogenous spreading of the infection.^4^ Primary pituitary gland TB is a sporadic condition, and only <110 cases have been reported till now.^1^ ^-^ ^65^ We present a case of a 50-year-old woman with the radiological diagnosis of the pituitary gland enlargement, suspected to be a macroadenoma. However, this case was given turn into pituitary TB. We report this case because misdiagnosis and wrong treatment may result in in permanent endocrine dysfunction.

## CASE REPORT

A 50-year-old woman presented to the neurosurgery outpatient department complaining of progressively increasing headache, and diminished vision for 1 year. On physical examination, the patient was conscious and oriented. There was no pallor, icterus, cyanosis, clubbing, edema or lymphadenopathy on clinical examination. Her weight and height were 164 cm and 72 kg respectively. Her Glasgow coma score was 15 (E4V5M6). The lungs examination was clear on auscultation. The patient was hypertensive on medication. The routine laboratory workup showed hemoglobin of 10.6 g/dl (reference range [RR]; 14g/dl – 16g/dl), leukocytes 8300/mm^3^ (RR; 4000 – 11000/mm^3^), and platelets 2.2x10^5^/mm^3^ (RR; 1.5x10^5^– 4.5x10^5^/ mm^3^). ESR was 40 mm in the first hour. The biochemical examination showed prolactin level of 58.00 ng/ml (RR; 2.1 - 17.7 ng/ml), luteinizing hormone <0.216mIU/ml (RR; 1.5 - 9.3 mIU/ml), follicle-stimulating hormone 3.07 mIU/ml (RR; 1.4 - 18.1 mIU/ml), serum cortisol 2.27 ng/ml (RR; 4.30 - 22.0 µg/dl), thyroid-stimulating hormone 0.2µIU/ml (RR; 0.35 - 5.5 IU/ml), T3 – 0.96 ng/dl (RR; 0.6 - 1.65 ng/ml), and T4 - 4.30 µg/dl (RR; 4.4 -11 µg/dl). The serum electrolytes were within normal limits. Her chest X-ray revealed normal lung parenchyma. There was no family or close contact history with tuberculosis. No evidence of extrapulmonary lesion that could raise the suspicion of tuberculosis was noted. The contrast-enhanced magnetic resonance imaging (MRI) scan revealed enhancing mass lesion of 1.7 x 1.4 cm arising from the sella, causing compression of optic chiasma (Figure 11B).

**Figure 1:**
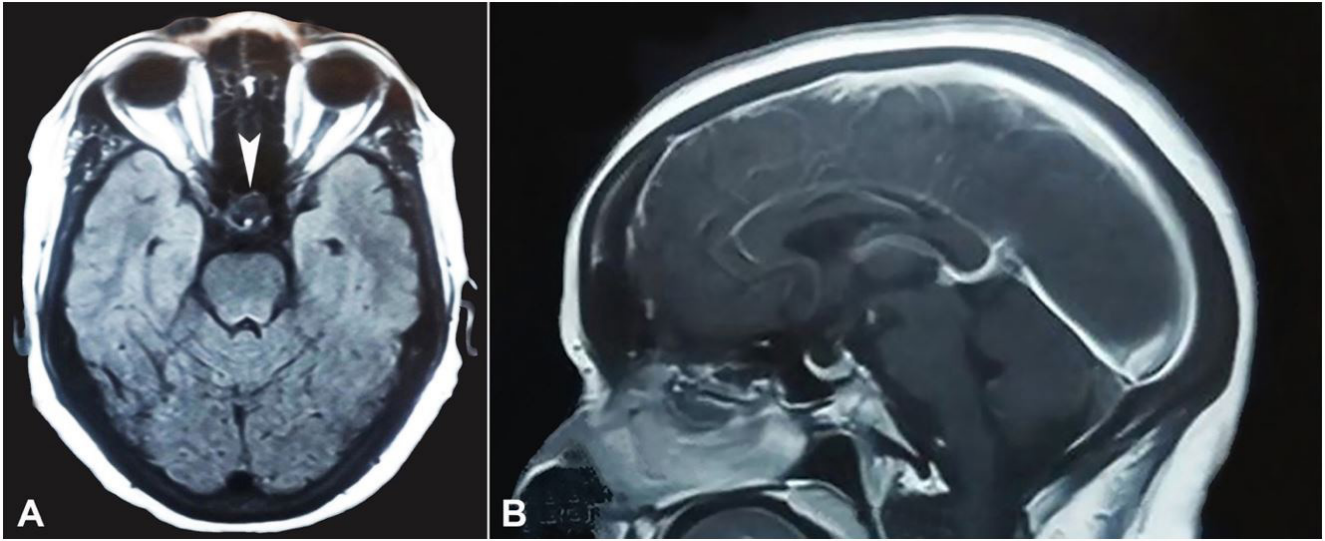
Brain MRI. **A** – T1W axial, and **B** – Sagittal Plane - Strongly enhancing a mass lesion measuring 1.7 x 1.4 cm arising from supra sellar region causing compression over optic chiasma without intrasellar or parasellar extension (arrowhead).

**Figure 2:**
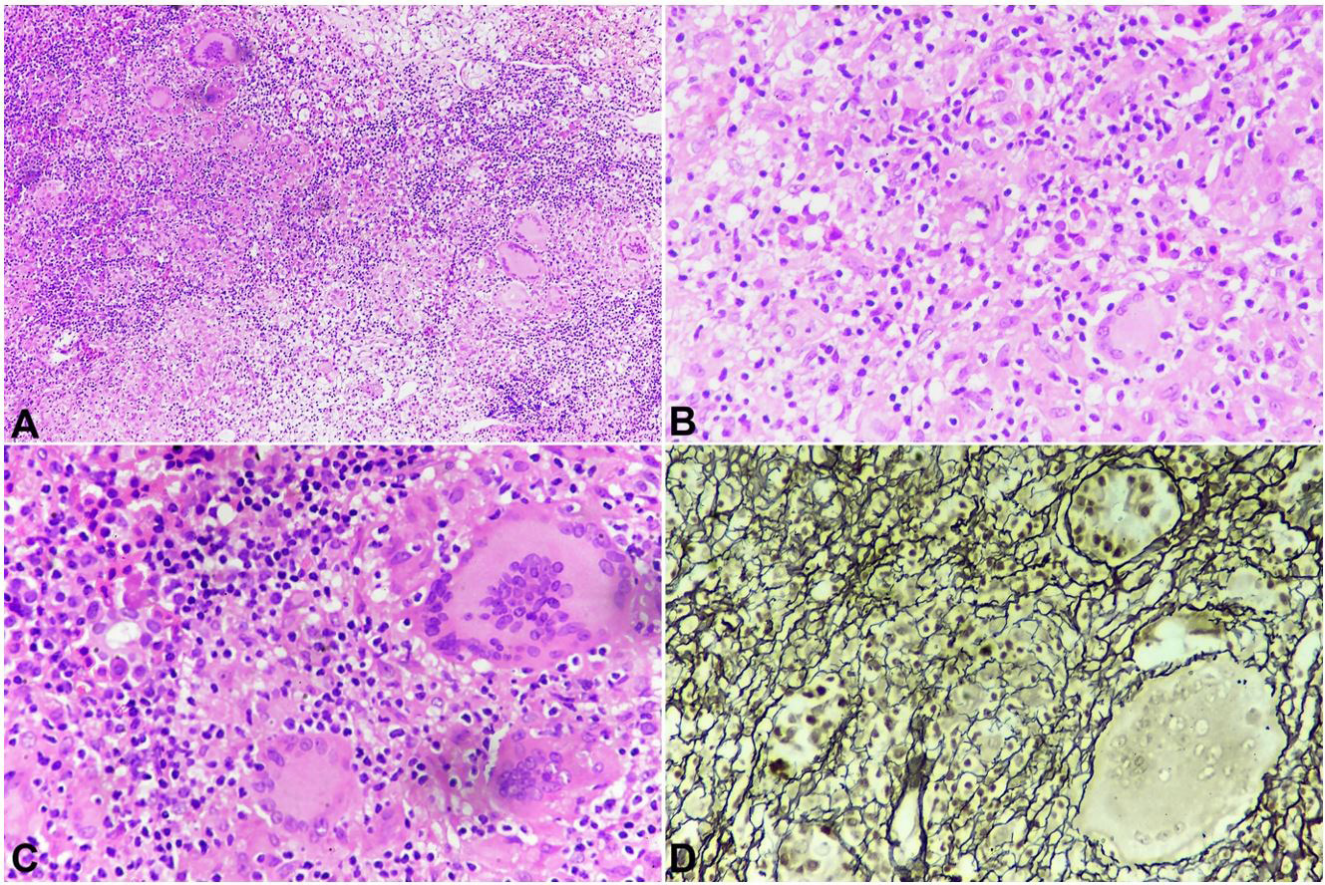
Photomicrograph of the pituitary gland. **A** – Many well-formed epithelioid granulomas (H& E: 100x); **B** – Well-formed epithelioid granulomas, Langhans giant cells, along with pituitary parenchymal cells (H&E: 400x); **C** – Granulomas with pituitary parenchymal cells (H& E: 400x); **D** – Reticulin stain: Well preserved pituitary acinar structure (400X).

**Figure 3:**
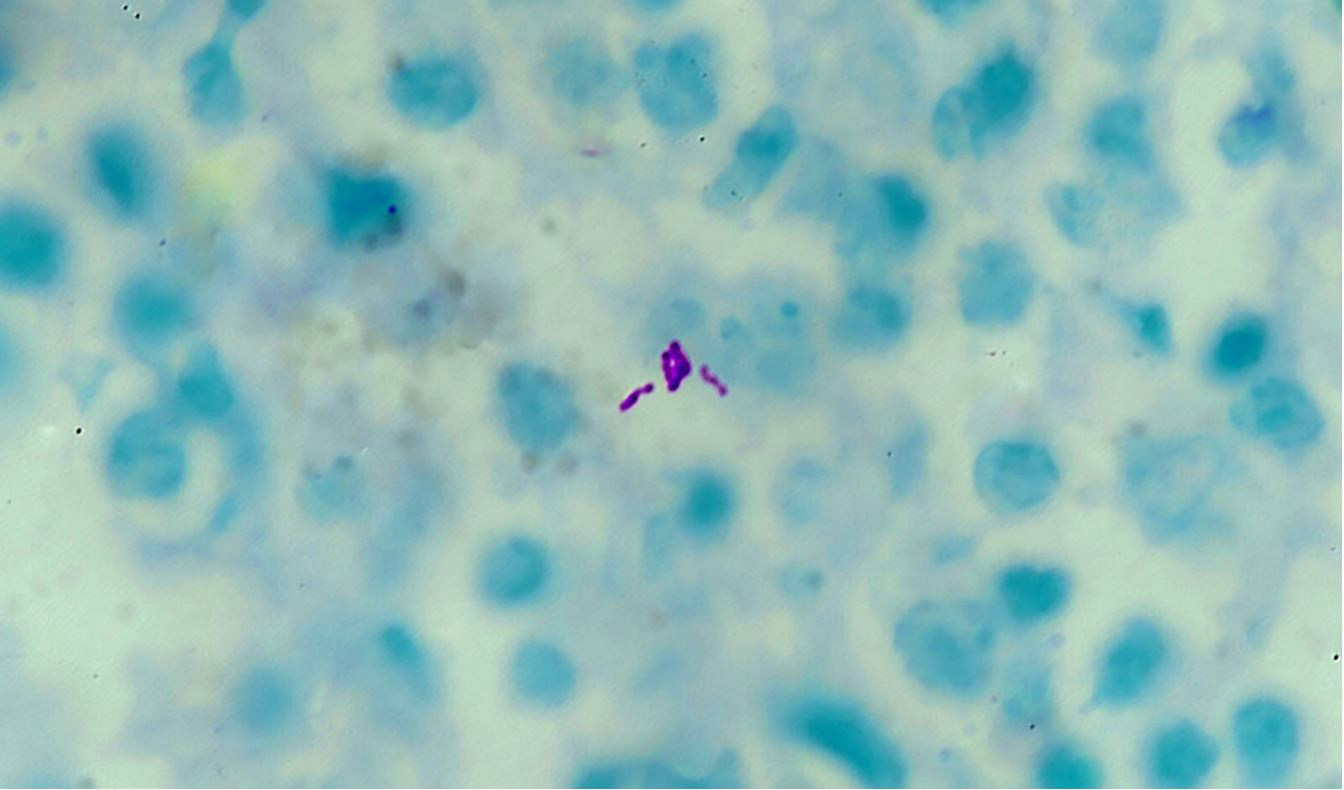
Photomicrograph of the pituitary gland showing acid-fast bacilli (Ziehl Neelsen stain, 1000x).

A radiological diagnosis of pituitary macroadenoma was given, and the patient was submitted to a transsphenoidal endonasal tumor excision. The postoperative period was uneventful. The histopathology showed numerous epithelioid cell granulomas with Langhans giant cells with a mixed and variable proportion of pituitary parenchymal cells arranged in a nested and alveolar pattern (Figure 222C). Reticulin stain demonstrated reticulin fibers around the nest of viable pituitary parenchymal cells (Figure 2D). Ziehl Neelsen staining demonstrated acid-fast bacilli (Figure 3).

Based on the above histomorphology, a diagnosis of Pituitary tuberculoma was given. Suspicion of pituitary adenoma was ruled out by the reticulin stain, and the demonstration of acid-fast bacilli by Ziehl Nelson stain. Treatment for tuberculosis was initiated with a 2-month combination of isoniazid, rifampin, pyrazinamide, and ethambutol, followed by 7 months of isoniazid and rifampicin. Her headache rapidly resolved and restored normal vision. Her hormone levels returned to normal after 2 months of follow-up. The outcome was uneventful and the patient is doing well after 15 months of follow-up.

## DISCUSSION

In the absence of pulmonary TB or other organ involvement, the primary pituitary TB is a very uncommon disease. Only less than a hundred and ten cases of primary pituitary TB are reported in the literature.^1^ ^-^ ^65^ We retrieved 106 cases of pituitary tuberculosis in a literature review from 1924 to 2019, using the keywords primary tuberculosis, pituitary, Ziehl Nelsen stain, and acid-fast bacilli at PubMed. Out of 106 cases, 51 cases were reported from India. The age ranged from 5 years to 69 years. The mean age of the cases was approximately 35.41 years, and the median age was 35 years. The females accounted for 71.84% (74/103) cases, and males accounted for 28.16% (29/103). In three cases, gender was not mentioned. The females: males ratio was 2.55:1. The history of tuberculosis or close contacts was reported only in 35 cases .^6^ ^,^ ^7^ ^,^ ^12^ ^,^ ^14^ ^,^ ^16^ ^,^ ^17^ ^,^ ^20^ ^,^ ^21^ ^,^ ^24^ ^,^ ^27^ ^,^ ^33^ ^,^ ^35^ ^,^ ^37^ ^,^ ^41^ ^,^ ^44^ ^,^ ^49^ ^,^ ^52^ ^,^ ^56^ ^,^ ^58^ ^,^ ^59^ ^,^ ^64^

The intracranial tuberculomas can occur at any age and commonly affects the young adult.^4^ ^,^ ^5^ It accounted for 30 – 34% of all intracranial space-occupying lesions before the emergence of antitubercular drugs.^5^ However, currently, it comprises 0.15–4% of all intracranial space-occupying lesions.^6^ The majority of pituitary TB cases were reported from the Indian subcontinent probably due to the high prevalence of tuberculosis in this country.^6^ The primary pituitary TB may be diagnosed in patients from 8 to 68 years (mean age of 34.1±13.6 years), and females are more affected than males.^4^ The hematogenous spread and extension from tubercular infection of paranasal sinuses have been suggested in the literature.^3^ However, the route of primary pituitary infection by the TB bacilli is still unclear.^3^ Headache, visual disturbances, low-grade fever, and vomiting are common clinical symptoms.^3^ ^,^ ^6^ Endocrine symptoms such as galactorrhea & amenorrhea are seen in females.^4^ ^,^ ^6^ Polyuria and polydipsia may also be present.^4^ ^,^ ^5^ The presence of central diabetes insipidus is a clue that may help to differentiate the pituitary TB from the pituitary adenoma.^4^ However, the misdiagnosis of pituitary tuberculoma as pituitary adenoma is common and may lead to irreversible endocrine dysfunction.^4^ The anterior pituitary hypofunction and hyperprolactinemia are common endocrine dysfunctions in pituitary TB.^4^ In our case, the patient presented with complaints of progressively increasing headache and diminished vision for 1 year without neurological deficit. Magnetic Resonance Imaging (MRI) is the foremost radiological technique for diagnosing and making the differential diagnosis of pituitary lesions.^3^ ^,^ ^4^ ^,^ ^7^ However, based on the MRI findings, it can be very difficult to differentiate between pituitary tuberculoma and adenoma.^3^ ^,^ ^4^ ^,^ ^7^ The thickening and nodularity of the pituitary stalk are considered useful signs to differentiate pituitary tuberculoma from an adenoma.^3^ ^,^ ^7^ However, this sign is non-specific and is also present in other inflammatory and neoplastic lesions of the pituitary gland such as syphilis, sarcoidosis, idiopathic hypophysitis, Wegner’s granulomatosis, neurocysticercosis, and lymphomas. The pattern of the enhancement is a useful tool in differentiating tuberculomas from other pituitary lesions.^3^ The lesion may have a hyperintense appearance on the T2 weighted image or may appear as a hyperintense center surrounded by a hypointense rim with peripheral ring enhancement of the lesion and enhancement of the adjacent dura and basal cistern.^7^ The tubercular caseation appears as a non-enhancement area.^7^ The magnetic resonance spectroscopy (MRS) detects the specific chemicals in tissues of interest.^4^ The caseous necrosis of tuberculoma demonstrates the lipid resonance at 0.9 and 1.3ppm.^4^ However, these MRS findings can be found in lymphoma and toxoplasmosis.^4^ In the present case, MRI favored the diagnosis of a pituitary adenoma. The pituitary adenoma was the commonest misdiagnosis for pituitary TB reported in the literature, and therefore histological confirmation is the main diagnostic modality.^3^ ^,^ ^4^ ^,^ ^7^ The transsphenoidal surgery is the better route for the diagnosis confirmation and decompression of adjacent structures preventing contamination of the intracranial structures.^4^ The histopathological examination reveals epithelioid cell granulomas, Langhans giant cells and caseous necrosis that may be occasional.^3^ ^,^ ^4^ ^,^ ^7^ The demonstration of the *Mycobacterium tuberculosis* can be done by culture, Ziehl Neelsen stain, or polymerase chain reaction and is usually confirmatory.^3^ ^,^ ^4^ The conservative management of pituitary TB may be possible if other tests confirm the diagnosis pre-operatively, such as cerebrospinal fluid PCR for tuberculosis in cases with co-existing tuberculous meningitis.^4^ The early diagnosis and prompt use of anti-tubercular drugs result in a better prognosis. In contrast, a delayed diagnosis could lead to permanent endocrine dysfunction.^3^ The anti-tubercular drugs that cross the blood-brain barrier are given to patients for 9 to 24 months depending on the clinical and imaging outcome.^3^ ^,^ ^4^ ^,^ ^7^

## CONCLUSION

The non-specific radiological findings challenge the pre-operative diagnosis of primary pituitary TB. However, a high clinical suspicion, especially in endemic regions, can minimize unnecessary invasive procedures and surgical interventions. Primary pituitary tuberculosis should be included in the differential diagnosis of sellar lesions to avoid unnecessary surgical interventions. The early clinical suspicion and prompt use of anti-tubercular drugs help to prevent irreversible endocrine dysfunction. We report this rare case of pituitary TB with the demonstration of acid-fast bacilli.
